# The Interfacial Structure and Bonding Properties of the Al(111)/CrB_2_(0001) Interface: Insights for Advanced Al-Based Composites

**DOI:** 10.3390/nano15070529

**Published:** 2025-03-31

**Authors:** Jingwen Sun, Mingjie Wang, Ben Wang, Zhongxian Chen

**Affiliations:** 1 School of Intelligent Manufacturing, Huanghuai University, Zhumadian 463000, China; sjw@huanghuai.edu.cn (J.S.); bw@huanghuai.edu.cn (B.W.); zhongxian1984@163.com (Z.C.); 2 School of Materials Science and Engineering, North University of China, Taiyuan 030051, China

**Keywords:** first-principles calculation, Al(111)/CrB_2_(0001) interface, interfacial stability, electron structure, doped interface

## Abstract

This research focuses on the structural and bonding characteristics of the Al(111)/CrB2(0001) interface, aiming to clarify the adhesion mechanisms of CrB_2_ coatings on aluminum composites. Utilizing first-principles calculations grounded in density functional theory (DFT), we systematically examined the interfacial properties of both clean and doped Al(111)/CrB_2_(0001) systems. And key aspects such as binding energy, electron density distribution, and chemical bonding types were thoroughly evaluated. The results demonstrate that the Cr-terminated HCP stacking arrangement at the Al(111)/CrB_2_(0001) interface achieves the maximum adhesion work and minimal interfacial energy. This is primarily due to the strong covalent interactions between Al-p and Cr-p orbitals, which contribute to exceptional interfacial strength and stability. Furthermore, the incorporation of Fe, Mg, and Mn at the interface not only markedly improves working adhesion but also effectively lowers the interfacial energy for the Cr-terminated HCP stacking configuration. This phenomenon significantly enhances the overall bonding performance of the Al/CrB_2_ system. Conversely, the addition of Cu, Zn, and Si leads to an increase in interfacial energy, negatively impacting the bonding quality. Analysis of binding energies at the doped interface revealed a consistent trend among the elements: Fe > Mn > Mg > Si > Zn > Cu. These findings offer valuable guidance for the design and optimization of Al-based surface coatings with improved performance.

## 1. Introduction

Aluminum-based alloys play a crucial role in modern industrial applications due to their exceptional combination of low density, excellent mechanical properties, and outstanding electrical conduction characteristics [1]. Specifically, in the automotive, aerospace, and electronics sectors, the expanding use of aluminum alloys markedly enhances product performance and reduces energy consumption [2]. However, the surface properties, particularly wear and corrosion resistance, of Al alloys require further enhancement. Researchers have explored various surface treatment methods, such as coatings, heat treatments, and surface modifications, to enhance these properties [3]. The traditional superhard coatings require high-temperature and high-pressure preparation conditions. In addition, the transition metal boride coatings are expected to be more widely used due to their strong covalent bonds and rich electronic structure. Since Cr is an excellent corrosion-resistant element, CrB_2_ coatings are expected to meet the needs of offshore equipment for good mechanical properties and excellent electrochemical performance. The ceramic material CrB_2_, known for its exceptional wear resistance and chemical stability, significantly enhances the surface performance as a coating [4,5]. Despite existing studies demonstrating CrB_2_‘s potential to enhance wear and corrosion resistance [6,7,8,9], these studies have primarily concentrated on macroscopic performance tests and coating preparation methods. The fundamental aspects of atomic-level interactions occurring at the interface of CrB_2_ coatings with aluminum-based substrates have not yet been thoroughly investigated, leaving significant gaps in our knowledge of these critical surface phenomena. These interfacial characteristics are vital for the long-term stability and protection effectiveness of the coatings [10]. Consequently, systematically studying the physicochemical properties of Al/CrB_2_ interfaces can unveil the microscopic bonding mechanisms, bearing significant scientific value and wide-ranging applications for advance Al composites.

First-principles calculations, grounded in density functional theory (DFT), serve as a potent instrument for probing into the microstructure and properties of materials [11,12]. This methodology is adept at forecasting the electronic structure, energy states, and geometric configurations of materials from a quantum mechanical standpoint, thus uncovering the inherent characteristics of materials [13,14,15,16]. This investigation employs density functional theory-based computational methods to systematically examine the fundamental characteristics of Al/CrB_2_ interfacial systems, including bonding energy parameters, electronic charge distribution patterns, and chemical interaction mechanisms. Through simulation of the atomic arrangement and electron density at the interface, this research elucidates the interactions between the CrB_2_ coating and the aluminum alloy substrate at an atomic level. Such insights are crucial for the optimization of coating performance and the enhancement of surface protection for aluminum alloys.

In Al-based composite materials, alloying elements like silicon and zinc, besides aluminum [17,18,19,20], demonstrate substantial doping effects at the Al(111)/CrB_2_(0001) interface, which significantly influences the coating–substrate interfacial characteristics. Elemental doping has the ability to modulate the interfacial electronic structure and chemical bonding, which in turn affects the adhesion properties of the coating and the overall properties of the composite material [21,22,23]. Although empirical methods encounter limitations in investigating dopant influences, computational simulations based on fundamental principles offer a robust analytical framework for such studies [24,25,26,27].

To gain deeper insights into the characteristics of the Al(111)/CrB_2_(0001) interface, we employed a first-principles computational framework to analyze its interfacial properties and assess the effects of typical alloying elements (Fe, Mg, Zn, Si, Mn, and Cu) commonly found in aluminum alloys. The conclusions drawn from these simulations are contingent on the assumption of dilute doping within the minimal unit cell framework. In this work, adhesion work, interfacial energy, and electronic structure were systematically calculated for the Al(111)/CrB_2_(0001) system using first-principles techniques. Furthermore, the influence of these alloying elements on the interfacial energy and electronic behavior of the Al(111)/CrB_2_(0001) interface was simulated. The primary objective was to establish a detailed understanding of the structural compatibility between the anisotropic CrB_2_ crystal planes and the face-centered cubic Al lattice, as well as to evaluate the stability of the interfacial configuration.

## 2. Calculation Methods and Models

### 2.1. Methods

In this paper, aiming to comprehensively analyze the physicochemical properties encompassing the bulk, surface, and interfacial characteristics of the Al and CrB_2_ models, we utilized the CASTEP software package for performing calculations grounded in density functional theory (DFT) [28]. The computational framework implemented the Generalized Gradient Approximation (GGA) methodology, integrating the Perdew–Burke–Ernzerhof (PBE) exchange–correlation functional to simulate electron exchange–correlation phenomena [29]. Furthermore, to ensure computational accuracy while simultaneously reducing the requisite computational resources, ultra-soft pseudopotentials were employed. Throughout the geometric optimization process, the BFGS algorithm was utilized [30], and the achievement of the ground state was facilitated by resolving the Kohn–Sham equations, employing the self-consistent field (SCF) procedure [31,32]. In an effort to minimize the mutual influence among model layers, a vacuum spacer layer, 15.0 Å in thickness, was incorporated within the models concerning surfaces or interfaces [33,34]. This strategic design enables a more precise study and analysis of the characteristics pertaining to surfaces or interfaces.

To ensure the accuracy of the calculations, we carefully selected and adjusted the computational parameters. The k-point mesh for Al bulk was set to 10 × 10 × 10, and for CrB_2_ bulk, it was set to 12 × 12 × 12. For the surface and interface, the k-point meshes were set to 16 × 16 × 1 and 18 × 18 × 1, respectively, with a uniform cutoff energy of 500 eV, as shown in Table 1. Additionally, for the surface and interface structures, the convergence criterion for the self-consistent field (SCF) was set to 1.0 × 10^−6^ electron volts per atom. The computational simulations were conducted with a convergence criterion set at 5 × 10^−7^ eV/atom for the total energy difference and a force convergence threshold of 0.03 eV/Å.

### 2.2. Models

**Figure 1:**
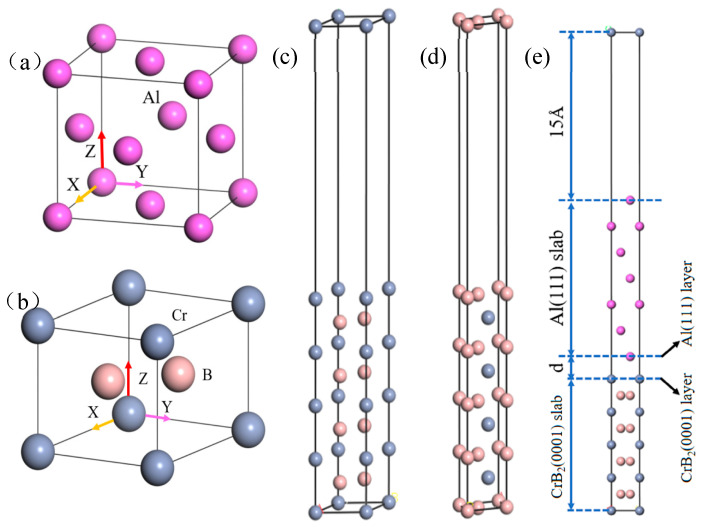
Bulk, surface, and interface structures of Al/CrB_2_: (**a**) Al bulk, (**b**) CrB_2_ bulk, (**c**) Cr-terminated CrB_2_(0001) slab, (**d**) B-terminated CrB_2_(0001) slab, (**e**) Al(111)/CrB_2_(0001) interface structure.

The bulk crystal configurations of Al and CrB_2_ are shown in Figure 1. The structure of Al is characterized as face-centered cubic (FCC), noted for its relatively expansive lattice constant and dense atomic packing [35]. Within the FCC structural variants, the (111) plane is distinguished by its tightly packed atoms. Conversely, CrB_2_ exhibits a hexagonal crystal structure. An experimental study by Weng Jiahao [36] identified the (0001) plane of CrB_2_ as its densest atomic plane, analogous to Al(111) in exhibiting a comparably low surface energy. The lattice dissonance between the Al(111) and CrB_2_ (0001) slabs is at 4.1%, which is confined within the cohesive stability threshold of less than 6% as postulated by Bramfitt [37]. Such correspondence suggests a propensity for these materials to form a hetero-structure with enhanced stability.

Table 2 shows essential crystallographic parameters for the Al bulk and CrB_2_ bulk, including lattice constants, formation enthalpy (ΔH*_f_*), and unit cell volume. The calculated results exhibit a considerable level of concordance with the empirical experimental data, not only corroborating the validity and precision of the computational methodologies applied in our investigation but also suggesting that the discrepancies between experimental settings and computational paradigms exert a negligible effect on the outcomes. Notably, the formation enthalpy, a key thermodynamic parameter for evaluating crystalline stability as discussed in reference [38], was precisely calculated for CrB_2_ in this investigation. The expression utilized for the calculation of the formation energies for CrB_2_ is delineated below:

(1) $$\Delta H_{f}=E_{CrB_{2}}^{Bulk}-E_{Cr}^{Bulk}-2E_{B}^{Bulk}$$

where $\Delta H_{f}$ represents the formation enthalpy of CrB_2_, $E_{\mathrm{Cr}B_{2}}^{\mathrm{bulk}}$ is the total energy of the CrB_2_ unit cell, and $E_{Cr}^{bulk}$ and $E_{B}^{bulk}$ represent the individual atomic energies of cubic Cr and α-B, respectively.

Selecting an optimal number of slab layers is pivotal for accurately simulating the bulk properties of materials, as referenced in [43]. While augmenting the number of layers can increment the precision of the simulation, it simultaneously escalates the computational burden. In this context, we evaluated the surface relaxation of the CrB_2_(0001) slab to ascertain the appropriate slab thickness by employing models incorporating various numbers of atomic layers. In particular, the CrB_2_(0001) surface exhibits two distinct terminations of the surface atomic layers. Our investigation exhaustively explores both termination types: Cr-termination and B-termination. Figure 1 graphically delineates these termination strategies. A 15 Å vacuum layer was incorporated above the Al(111) surface to replicate realistic interfacial conditions during the construction of the Al(111)/CrB_2_(0001) interface model, as illustrated in Figure 1.

While the 1 × 1 surface cell was employed to model doping effects at the Al(111)/CrB_2_(0001) interface, it is important to acknowledge the inherent limitations of minimal unit cells in DFT-based studies. Small cell sizes may restrict the ability to fully capture long-range structural relaxations and electronic interactions induced by dopants, potentially leading to oversimplified predictions of doping effects. This limitation has been extensively discussed in the literature, particularly in systems where dopant concentration or spatial distribution plays a critical role [44]. Nevertheless, the current approach provides a computationally tractable framework for preliminary screening of alloying element effects, and the conclusions should be interpreted within this context.

## 3. Results and Discussion

### 3.1. Surface Properties

The relative percentage change in interlayer spacing can be quantified by the following formula:

(2) $$\Delta d_{i,i+1}=\frac{d_{i,i+1}-d_{0}}{d_{0}}\times 100\%$$

where *d*_0_ represents the interlayer spacing of the crystal structure before relaxation, and $d_{i,i+1}$ represents the interlayer spacing after relaxation between the *i*th and (*i* + 1)th layers. $\Delta d_{i,i+1}$ represents the relative percentage change in the interlayer spacing.

We adopted the computational method proposed in references [45,46,47], enabling us to effectively calculate and evaluate the surface energy. The formula for calculating the surface energy of CrB_2_(0001) is as follows:

(3) $$E_{surface}=\frac{1}{2A_{surface}}\left[E_{slab}-N_{B}\mu_{B}^{slab}-N_{Cr}\mu_{Cr}^{slab}+PV-TS\right]$$

The calculations were performed at 0 K, with the TS term being zero. In the condensed phase, the PV term is negligible. Hence, Equation (3) can be simplified as follows:

(4) $$E_{surface}=\frac{1}{2A_{surface}}\left[E_{slab}-N_{B}\mu_{B}^{slab}-N_{Cr}\mu_{Cr}^{slab}\right]$$

In this context, $E_{surface}$ denotes the CrB_2_(0001) surface energy, $A_{surface}$ corresponds to the CrB_2_(0001) surface area, $E_{\mathrm{slab}}$ signifies the total energy of the surface system, $N_{B}$ and $N_{\mathrm{Cr}}$ indicate the quantities of B and Cr atoms within the model, respectively, and $\mu_{B}^{\mathrm{slab}}$ and $\mu_{\mathrm{Cr}}^{\mathrm{slab}}$ symbolize the chemical potentials of B and Cr atoms in the surface system, respectively.

To validate the reliability of the simulations and accurately capture surface properties, convergence analyses were performed by evaluating surface energy and the relative variation in interatomic layer distances. The corresponding computational outcomes are presented in Table 3 and Table 4.

Analysis of the percentage changes in interlayer spacing subsequent to the optimization of the CrB_2_(0001) surface model elucidates that relaxation predominantly impacts the uppermost three layers, with this effect being notably augmented by both the slab thickness and the methodology employed for surface termination. This phenomenon manifests uniformly across varying layers within the model and irrespective of the termination technique utilized, highlighting a consistent pattern where alterations in the outer layers are markedly more evident in comparison to those in the inner layers. From Table 3, Cr-terminated CrB_2_(0001) surfaces display a diminished relative displacement amidst adjacent atomic layers during the relaxation phase compared with those of B-terminated CrB_2_(0001) surfaces, which suggests a reduced surface energy of Cr-terminated surfaces. All these results demonstrate a trend towards stabilization in interlayer spacing changes upon reaching a model thickness of nine layers, for both Cr- and B-terminations. This stabilization implies the convergence; therefore, for the purposes of analyzing surface stability, we employed a nine-layer model as a standard for calculating surface energy for both Cr- and B-terminations, as corroborated by the data in Table 4. Drawing parallels from the findings articulated in references [48,49], it is inferred that the surface structure of the Al(111) surface attains stability when the model comprises seven or more atomic layers. Leveraging this insight, the present study opts for a seven-layer atomic thickness as a foundational benchmark for constructing the Al(111) surface model, thereby ensuring an accurate representation of its inherently stable surface characteristics.

The chemical potential of a single atom and the bulk structure keep balance in the same system; thus,

(5) $$\mu_{CrB2}^{bulk}=\mu_{Cr}^{slab}+2\mu_{B}^{slab}$$

(6) $$\mu_{CrB2}^{bulk}=\mu_{Cr}^{bulk}+2\mu_{B}^{bulk}+\Delta H_{f}$$

where $\mu_{CrB2}^{bulk}$ represents the chemical potential of the CrB_2_(0001) bulk structure, while $\mu_{Cr}^{bulk}$ and $\mu_{B}^{bulk}$ denote the chemical potentials of the individual Cr and B atoms in the bulk structure, respectively. Further, $\mu_{\mathrm{Cr}}^{Slab}$ and $\mu_{B}^{Slab}$ represent the chemical potentials of the individual Cr and B atoms in the surface model, respectively.

Therefore, the surface energy of the CrB_2_(0001) slab can be written as the following formula:

(7) $$E_{surface}=\frac{1}{2A_{surface}}\left[E_{slab}-N_{Cr}\mu_{CrB2}^{bulk}+\left(2N_{Cr}-N_{B}\right)\mu_{B}^{slab}\right]$$

Combining Equations (5) and (6), the formation enthalpy of CrB_2_(0001) can be expressed by the following formula:

(8) $$\Delta H_{f}=\left(\mu_{Cr}^{slab}-\mu_{Cr}^{bulk}\right)+2\left(\mu_{B}^{slab}-\mu_{B}^{bulk}\right)$$

The difference between the surface chemical potential and the bulk chemical potential of a single element is defined as

(9) $$\Delta \mu_{Cr}=\mu_{Cr}^{slab}-\mu_{Cr}^{bulk},\;\Delta \mu_{B}=\mu_{B}^{slab}-\mu_{B}^{bulk}$$

To guarantee the structural stability of the surface model, the chemical potentials of the elements in the surface model and the bulk structure need to be satisfied with the following conditions:

(10) $$\mu_{Cr}^{slab}\leq \mu_{Cr}^{bulk},\;\mu_{B}^{slab}\leq \mu_{B}^{bulk}$$

Combining Equations (8)–(10), Equation (9) can be further organized as

(11) $$\frac{1}{2}\Delta H_{f}\leq \left(\mu_{B}^{slab}-\mu_{B}^{bulk}\right)=\Delta \mu_{B}\leq 0$$

By substituting Equation (9) into Equation (7), the calculation formula for the surface energy of CrB_2_(0001) can be rearranged as

(12) $$E_{surface}=\frac{1}{2A_{surface}}\left[E_{slab}-N_{Cr}\mu_{CrB2}^{bulk}+\left(2N_{Cr}-N_{B}\right)\left(\Delta \mu_{B}+\mu_{B}^{bulk}\right)\right]$$

**Figure 2:**
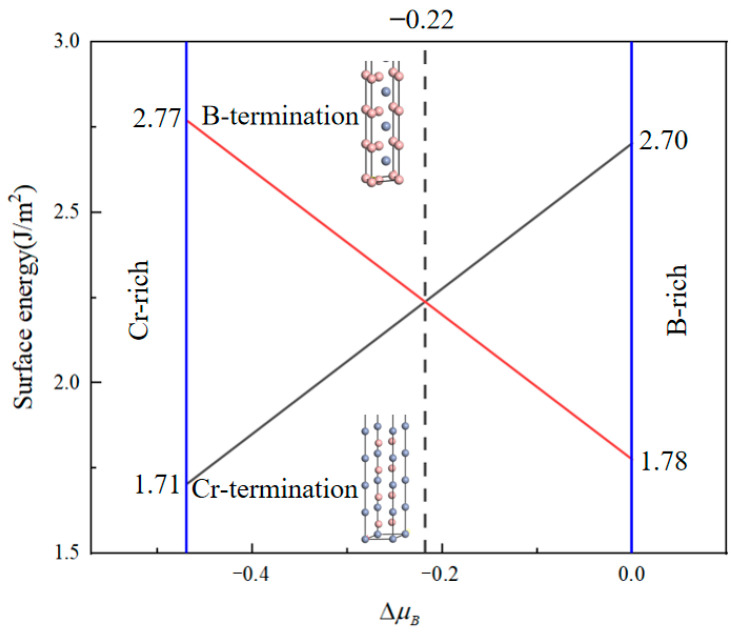
CrB_2_(0001) surface energy function of $\Delta \mu_{B}$.

Figure 2 illustrates the relationship between the surface energy of CrB_2_(0001) surface models with different terminations and the chemical potential difference. It is observed that the surface energy exhibits varying trends with changes in the chemical potential difference.

Specifically, within the chemical potential difference interval of −0.47 to 0 eV, there is a notable decrease in the surface energy of the B-terminated surface, descending from 2.77 J/m^2^ to 1.78 J/m^2^. Simultaneously, the surface energy of the Cr-terminated surface exhibits a contrary trend, increasing from 1.71 J/m^2^ to 2.70 J/m^2^. Typically, surface stability is inversely proportional to surface energy; thus, a decline in surface energy implies enhanced stability, whereas an elevation indicates augmented surface reactivity. In the specified range of the chemical potential difference from −0.47 to −0.22 eV, the B-terminated surface manifests elevated surface energy relative to the Cr-terminated surface, denoting heightened activity of the B-terminated surface under conditions favoring Cr. Conversely, as the chemical potential difference ascends, notably from −0.22 to 0 eV, an increase in the reactivity of the Cr-terminated surface coupled with an augmentation in the stability of the B-terminated surface is observed under B-rich conditions. These dynamics suggest that the Cr-terminated surface may possess superior stability across a wider spectrum of chemical potential differences compared to the B-terminated surface, potentially attributable to the diminished dipole moment of the positively charged ions present on the surface. Such insights elucidate the pivotal role of CrB_2_ particles within the nucleation process of materials, offering a nuanced understanding of surface interactions under varied chemical environments.

### 3.2. Interface Properties

**Figure 3:**
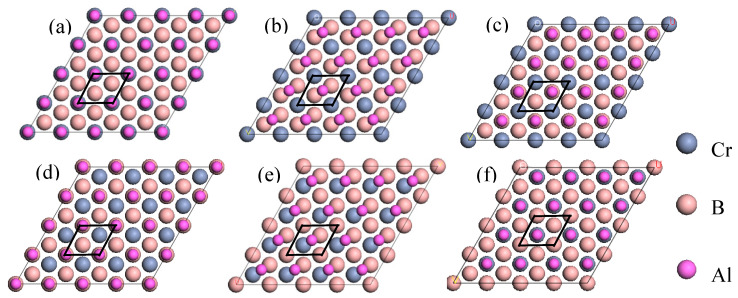
The locations of interfacial atoms of the Al(111)/CrB_2_(0001) interface: (**a**) Cr-terminated OT; (**b**) Cr-terminated MT; (**c**) Cr-terminated HCP; (**d**) Cr-terminated OT; (**e**) Cr-terminated MT; (**f**) Cr-terminated HCP.

Building upon the results derived from surface convergence and stability analyses, we developed an interface model of Al(111)/CrB_2_(0001). This model comprises a seven-layer Al(111) slab and a nine-layer CrB_2_(0001) slab. In addition, we examined three potential atomic stacking configurations for the CrB_2_(0001) slab on the Al(111) substrate, namely top (OT) stacking, middle (MT) stacking, and hexagonal close-packed (HCP) stacking. Specifically, for the OT stacking configuration, Cr or B atoms are aligned directly above the Al atomic layers, for the MT configuration, B or Cr atoms are situated between two adjacently positioned atoms in the Al layer, and for the HCP configuration, these atoms are aligned above the second layer of Al atoms. The variation in stacking modes significantly impacts the electronic structure and interatomic interactions at the interface. Accordingly, we constructed six distinct Al(111)/CrB_2_(0001) interface models, as depicted in Figure 3. This comprehensive modeling approach lays a robust theoretical basis for further detailed analysis and subsequent research endeavors.

**Figure 4:**
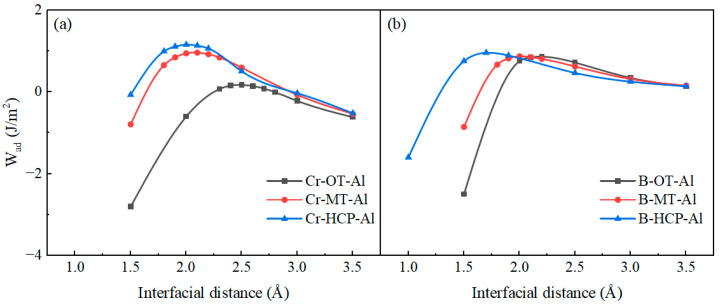
UBER curves for six different models of Al(111)/CrB_2_(0001) interface: (**a**) Cr-termination; (**b**) B-termination.

Furthermore, we evaluated the adhesion and stability of the interface by examining two critical metrics: the work of adhesion ($W_{ad}$) and the interfacial energy (γ_int_). These parameters quantitatively elucidate the adhesive characteristics and thermodynamic behavior of the interface. Preliminary computations of $W_{ad}$ were executed across a range of interface spacings without the optimization of the geometric model. The equations employed to calculate $W_{ad}$ and γ_int_ for the Al(111)/CrB_2_(0001) interface are delineated below:

(13) $$W_{ad}=\left(E_{total}^{Al}+E_{total}^{CrB2}-E_{total}^{Al/CrB2}\right)/A_{\mathrm{int}erface}$$

(14) $$\gamma_{\mathrm{int}}=E_{Al}^{slab}+E_{CrB2}^{slab}-W_{ad}$$

where $E_{total}^{Al}$ and $E_{total}^{CrB2}$ represent the total energies of the Al(111)/CrB_2_(0001) surfaces after relaxation, respectively, and $E_{total}^{Al/CrB2}$ denotes the total energy after the formation of the Al(111)/CrB_2_(0001) interface. $A_{\mathrm{int}erface}$ denotes the area of the interface, and $E_{Al}^{slab}$ and $E_{CrB2}^{slab}$ represent the surface energies of the individual Al(111) and CrB_2_(0001) surfaces, respectively. These calculations were conducted without the relaxation of atomic positions, an approach that aids in the more precise evaluation and determination of the optimal interface structure, while also significantly reducing the complexity of the calculations and the resources required. In the preliminary stage, the total energy of unoptimized interfacial configurations across multiple separation distances (*d*_0_) was determined through application of the Universal Binding Energy Relationship (UBER). This initial computational procedure generated optimized work of adhesion (*W_ad_*) and equilibrium separation parameters, providing benefits in computational resource optimization and cost reduction. Figure 4 demonstrates the adhesion energy variations among distinct interface terminations, delivering critical perspectives on the fundamental mechanisms governing interfacial bonding behavior.

Generally, smaller interfacial spacing correlates with stronger atomic bonding at the interface, resulting in a larger interfacial binding energy and, presumably, enhanced interfacial stability. The data clearly show that among the three stacking sequences, the hexagonal close-packed (HCP) configuration exhibits the smallest interfacial spacing and the largest interfacial binding energy. In most cases, reduced interatomic distances at the interface are associated with more robust bonding interactions between atoms, leading to increased binding energy and potentially improved stability across the interface. Experimental observations reveal that when comparing the three different atomic arrangements, the HCP (hexagonal close-packed) structure demonstrates the minimal interface separation distance along with the maximum binding energy at the interface.

Using the equilibrium separation distance (d_0_) determined from Figure 4 as a reference, a comprehensive relaxation procedure was applied to investigate multiple stacking arrangements of Cr- and B-terminated configurations. The interfacial distance derived from the UBER approach formed the foundation for subsequent structural refinement of the interface system. Following convergence to a stable configuration, the adhesion work and interfacial energy were calculated for the optimized structure. These quantitative outcomes are systematically summarized in Table 5.

From Table 5, one can see that the six interface models maintained consistency in interfacial spacing and exhibited an increase in adhesion work following relaxation. The measurements taken after complete relaxation more accurately reflect the system’s physical properties when it attains an equilibrium state. Furthermore, the stacking configuration of atoms at the interface significantly influences both the magnitude of adhesion work and the optimization of interfacial spacing. Specifically, interfaces featuring the HCP stacking mode displayed the highest adhesion energy. As the stacking mode transitioned from HCP to MT and subsequently to OT, a declining trend in adhesion energy was observed. This trend is partly attributed to the ability of interfaces with HCP stacking to achieve a stable configuration more swiftly during geometric optimization, thereby minimizing the lateral adjustments required by the interfacial atoms. For the interfaces characterized by OT and MT stacking modes, achieving energy minimization necessitates more complex, multi-step lattice adjustments. Concurrently, the stacking sequence of B atoms at the interface does not substantially influence the adhesion work, whereas the stacking arrangement of Cr atoms exerts a significant regulatory effect on the adhesion work. Therefore, among the six interfacial models investigated, the Cr-HCP-Al interface exhibits higher adhesion work, which is deemed the most stable.

**Figure 5:**
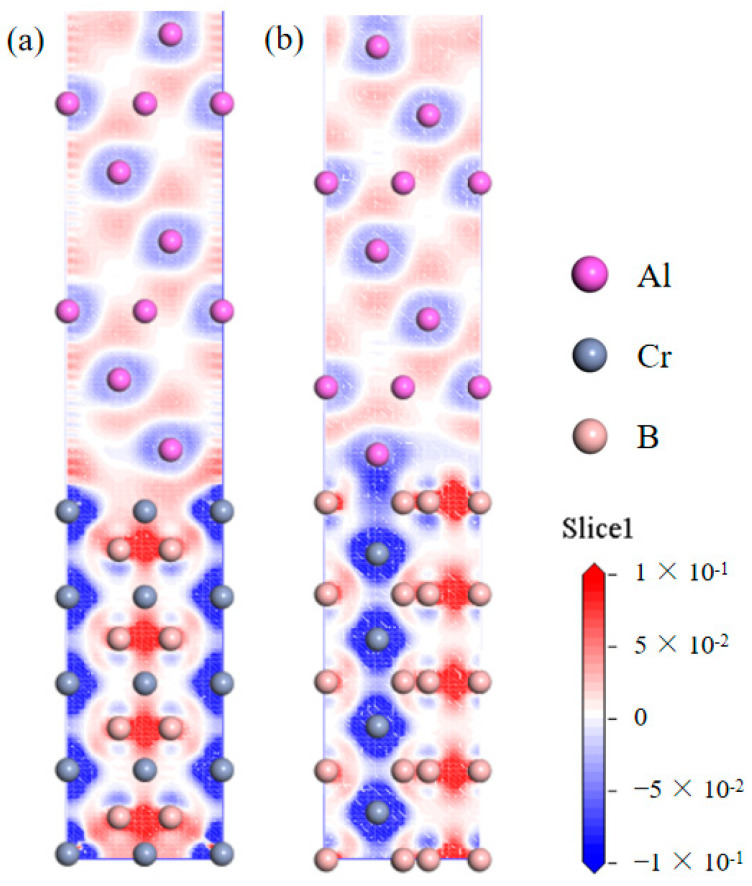
Charge density differences (Δρ) for (**a**) the Cr-terminated HCP interface and (**b**) the B-terminated HCP interface. Δρ is defined as the difference between the total charge density of the interface system and the sum of isolated Al(111) and CrB_2_(0001) slabs.

In pursuit of an advanced understanding of the electronic structure and chemical bonding characteristics at the Al(111)/CrB_2_(0001) interface, this investigation meticulously examined variations in charge density distribution. The charge density difference (Δρ) shown in Figure 5 was computed using the following formula:

Δρ = ρ_total_ − (ρ_Al slab_ + ρ_CrB2 slab_)

where ρtotal is the total charge density of the fully relaxed Al(111)/CrB_2_(0001) interface system. ρAl slab and ρCrB_2_ slab are the charge densities of the isolated Al(111) and CrB_2_(0001) slabs, respectively.

Figure 5 delineates the profiles of the charge density differential, effectively illustrating the distribution of charge across the relaxed Cr-terminated and B-terminated HCP interfaces. In Figure 5, regions displaying a negative value of valence electron density (depicted in blue) signify local electron scarcity, whereas regions marked by positive values (illustrated in red) denote local concentrations of electrons, which are generally indicative of enhanced interatomic bonding strength. The analysis of charge density differences elucidates that on the surface of Al(111), there exists a localized aggregation of electrons between Al atoms, culminating in the formation of an extensive electron cloud; this phenomenon suggests the establishment of robust Al-Al metallic bonds. Conversely, for the CrB_2_(0001) slab, the presence of a positive accumulation of electron density proximal to B atoms intimates the potential formation of covalent bonds among B atoms residing within the same layer. Furthermore, one can see that the valence electrons are transferred from Cr atoms to B atoms, concurrently with the manifestation of pronounced ionic bonds between contiguous atomic layers, thereby exerting a substantial impact on the electronic characteristics of the material. Specifically, as depicted in Figure 5a, the ionic bond formed between Cr and B atoms is notably stronger in comparison to the corresponding bond in Figure 5b. Additionally, the electron interactions within the first layer in Figure 5a are more conspicuous, signifying that the surface structure terminated by Cr exhibits a higher degree of stability relative to the B-terminated surface. This enhanced stability is presumably attributable to the augmented ionic bonding and electron localization at the Cr-terminated interface.

**Figure 6:**
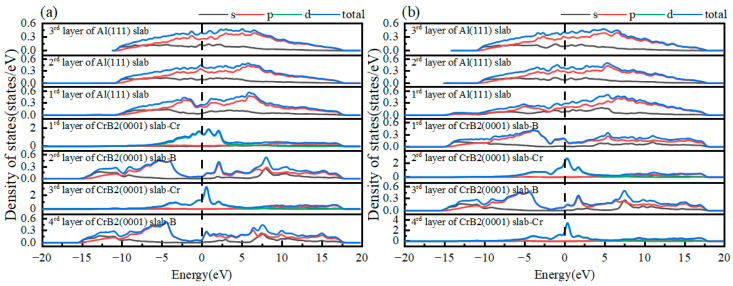
Partial density of states (PDOS) for atomic species in (**a**) the HCP-stacked interface with Cr-termination and (**b**) the HCP-stacked interface with B-termination.

This investigation employed partial density of states (PDOS) analysis to examine the electronic characteristics of the Al(111)/CrB_2_(0001) interface, providing insights into interfacial bonding mechanisms and charge transfer phenomena. Figure 6 presents the PDOS distributions for HCP-stacked interfaces with Cr- and B-terminations, where the Fermi level is marked by a vertical dashed line. Specifically, Figure 6a depicts the density of states (DOS) corresponding to Al-2p orbitals in the outermost Al(111) layer and Cr-3d orbitals in the surface layer of CrB_2_(0001). These states exhibit unique features distinct from bulk atoms, revealing hybridization between Al-2p and Cr-3d orbitals. Particularly, near the Fermi level, the PDOS of Cr-3d orbitals in the third CrB_2_(0001) layer shows a pronounced peak with substantially greater intensity compared to interior Cr atoms. This finding, along with the charge density differences presented in Figure 6a, indicates that the Cr-HCP-Al interface exhibits electronic properties consistent with metallic bonding characteristics.

Figure 6b reveals substantial orbital hybridization between Al-2p states in the surface layer of Al(111) and B-2p states in the outermost CrB_2_(0001) layer, occurring within the −14.5 to −10 eV energy range at the interface. This electronic interaction facilitates the development of robust covalent bonding, highlighting the sophisticated electronic coupling mechanisms and the complexity of interfacial bonding phenomena. Comparative examination of DOS profiles for interfacial and bulk Al atoms demonstrates reduced intensity and a lower energy shift in the DOS curve for surface Al atoms. This energy shift suggests electron donation from Al to neighboring B atoms, leading to nonpolar covalent bond formation. Supporting evidence is provided by the charge density difference analysis in Figure 5b, which displays enhanced 2p orbital occupancy for both Al and B atoms near the Fermi level, confirming covalent bonding at the interface. The PDOS results offer a comprehensive elucidation of the chemical bonding nature at the Al(111)/CrB_2_(0001) interface, providing an electronic structure-based explanation for the superior stability observed in Cr-terminated HCP configurations of the Al(111)/CrB_2_(0001) system.

### 3.3. Doping Interface Properties

**Figure 7:**
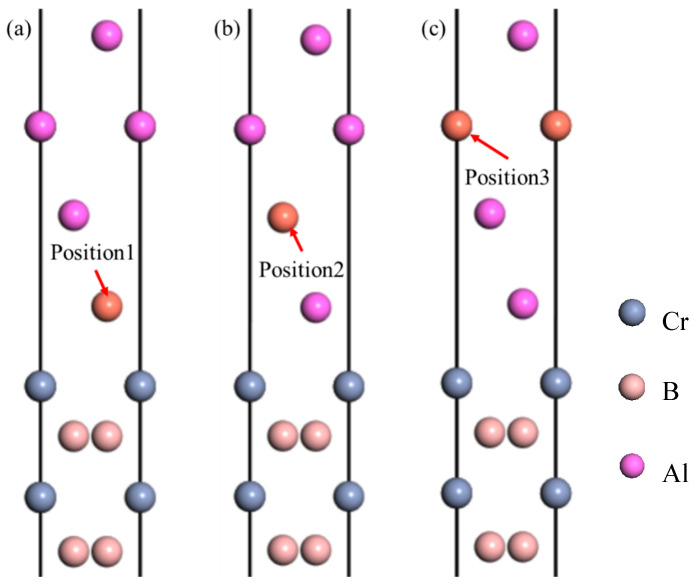
Alloying element incorporation at distinct atomic layers: (**a**) doping in the outermost Al(111) surface layer, (**b**) doping in the intermediate Al(111) layer, and (**c**) doping in the innermost Al(111) layer.

In the study presented here, we initially explored the pristine Cr-terminated HCP-stacked Al(111)/CrB_2_(0001) interface structure through computational simulations. This investigation was further expanded to assess the effects of doping with common aluminum alloying elements, including Cu, Mn, Si, Mg, Zn, and Fe, by substituting these elements for Al atoms at the interface. These alloying elements were strategically placed at various locations within the Al(111)/CrB_2_(0001) interface to evaluate and compare the adhesion work and interfacial energy associated with each configuration. Figure 7 demonstrates the designated doping locations: position 1 corresponds to atomic sites in the outermost Al(111) layer, position 2 to those in the intermediate layer, and position 3 to atomic sites in the innermost layer. For a thorough understanding of the effects of this doping, Table 6 provides a detailed analysis of the adhesion work and interfacial energy across these distinct doping positions, offering insightful perspectives on how different alloying elements can influence the interface characteristics in aluminum-based materials.

**Figure 8:**
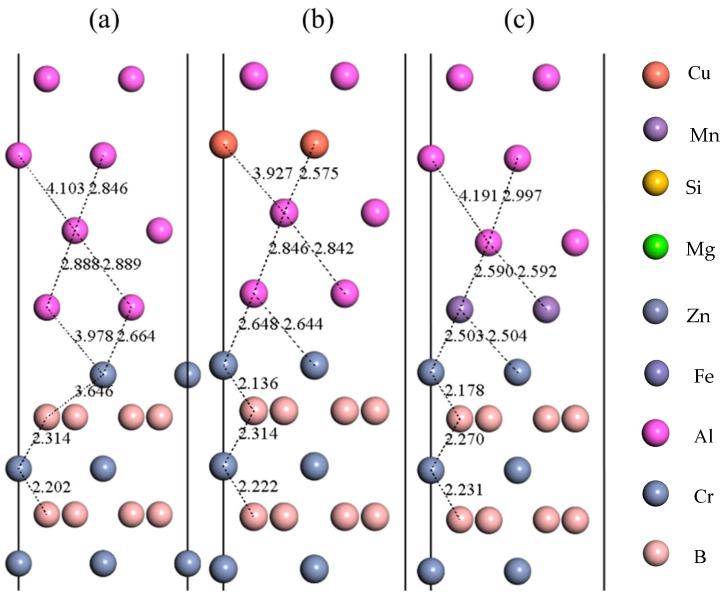

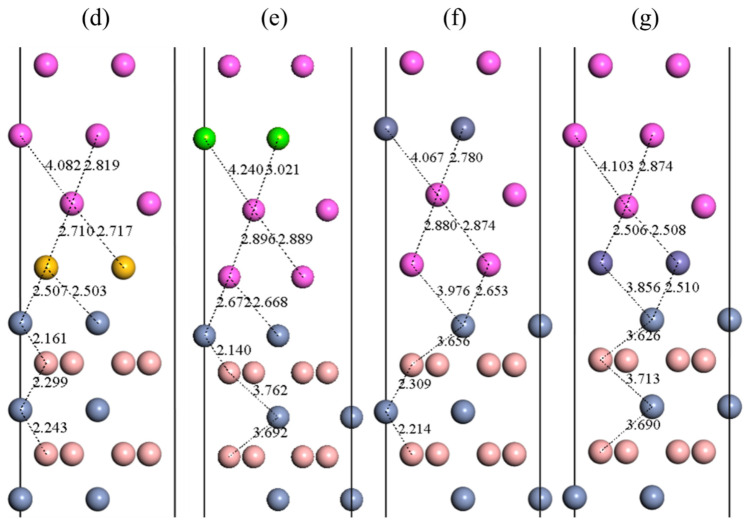
Relaxed interfacial configurations: (**a**) undoped system, (**b**) Cu-incorporated interface, (**c**) Mn-modified structure, (**d**) Si-doped system, (**e**) Mg-embedded interface, (**f**) Zn-substituted configuration, (**g**) Fe-integrated geometry.

The data in Table 6 delineate a clear pattern: the doping of Cu, Mg, and Zn at the third layer of the Al(111)/CrB_2_(0001) interface typically results in increased adhesion work compared to those in the upper layers, while simultaneously, the interfacial energy decreases. Specifically, doping with Cu and Zn at the third layer leads to reduced adhesion work values of 1.67 J/m^2^ and 1.73 J/m^2^, respectively, indicating a potential increase in interfacial energy for these configurations. Conversely, Mg doping at the same layer shows an adhesion work of 1.90 J/m^2^, higher than the undoped interface, suggesting a decrease in interfacial energy and enhanced adhesive strength of the coating. In cases where Mn, Si, and Fe serve as dopants, the atoms positioned at the first layer of the interface show an increase in adhesion work relative to the second and third layers, coupled with a reduction in interfacial energy. Remarkably, Si doping at the first layer maintains an adhesion work of 1.74 J/m^2^, consistent with the undoped interface, yet with a greater interfacial energy. Both Mn and Fe doping at the first layer exhibit elevated adhesion work, at 1.98 J/m^2^ and 2.15 J/m^2^, respectively, effectively lowering interfacial energy and reinforcing the adhesive properties. These outcomes suggest a hierarchy in the impact of doping elements on interfacial binding energy from highest to lowest: Fe > Mn > Mg > Si > Zn > Cu. The mechanisms behind increased adsorption work and subsequent structural relaxation at the interface are further illustrated in Figure 8.

Figure 8a delineates the undoped interface state, where Al atoms in the first layer of the Al(111) slab exhibit bond lengths ranging from 2.664 Å to 3.978 Å with Cr atoms during the relaxation process. Following the doping of the third layer with Cu, Mg, and Zn, the Al atoms in the first layer move closer to the Cr atoms, leading to a decrease in bond lengths post-relaxation. Doping the first layer with Mn, Si, and Fe results in these atoms approaching closer to the Cr atoms, forming Mn-Cr, Si-Cr, and Fe-Cr bonds with notably shorter lengths of 2.644 Å, 2.503 Å, and 2.510 Å respectively, compared to 2.664 Å in the undoped interface. The doping of the interface with alloy atoms such as Cu, Mn, Si, Mg, Zn, and Fe leads to varied lateral displacements among the atoms in the interfacial region when compared to the undoped state.

**Figure 9:**
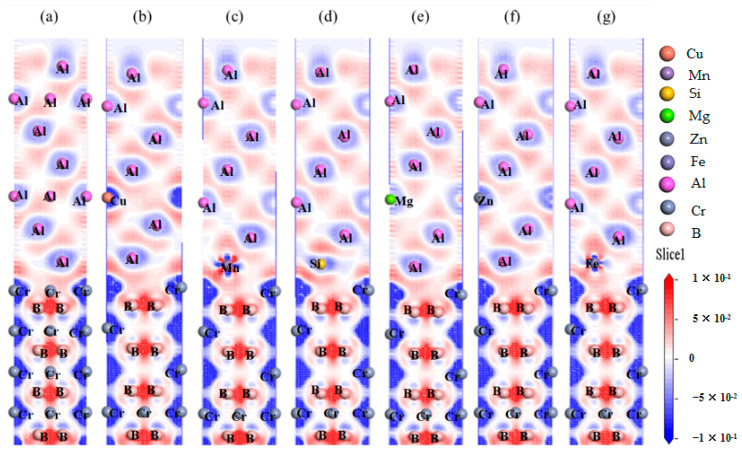
Charge density differences for doped interfaces: (**a**) clean, (**b**) Cu-doped, (**c**) Mn-doped, (**d**) Si-doped, (**e**) Mg-doped, (**f**) Zn-doped, and (**g**) Fe-doped systems. Δρ is computed relative to the pristine interface and isolated dopant atoms.

The electronic structure of both pure and doped interfaces is crucial for analyzing electronic bonding behaviors at these junctions. Figure 9 illustrates the charge density difference (Δρ) for doped interfaces, calculated by subtracting the charge densities of the pristine Al(111)/CrB_2_(0001) interface and the isolated dopant atoms from the total charge density of the doped system:

$$\Delta \rho_{\mathrm{doped}}=\rho_{doped\mathrm{int}erface}-\left(\rho_{pristine\mathrm{int}erface}+\rho_{dopand\mathrm{int}erface}\right)$$

This approach isolates the electronic perturbations induced by dopants. All geometries were fully relaxed prior to charge density calculations to account for structural adjustments caused by doping. In this plot, regions of charge concentration are indicated in red, while blue areas signify charge deficiencies. This pronounced charge transfer suggests localized accumulation and depletion of charge around the interfacial atoms. Notably, after Mn and Fe doping, there is a significant presence of local charge concentrations and deficiencies near the doped atoms, leading to a shared charge region between these and the Cr and B atoms. This is indicative of the formation of covalent bonds such as Mn-Cr, Mn-B, Fe-Cr, and Fe-B at the interface. In the Mn-doped interface, the substantial electron concentration and deficiency suggest stronger attractive interactions among the atoms. Conversely, interfaces doped with Cu and Zn display more extensive charge concentration areas, indicating diminished charge sharing and hence weaker interfacial bonding strength, particularly marked by a notable charge deficiency near Cu-doped atoms. Interfaces doped with Si and Mg show charge density differences that are largely consistent with those of the undoped interface.

**Figure 10:**
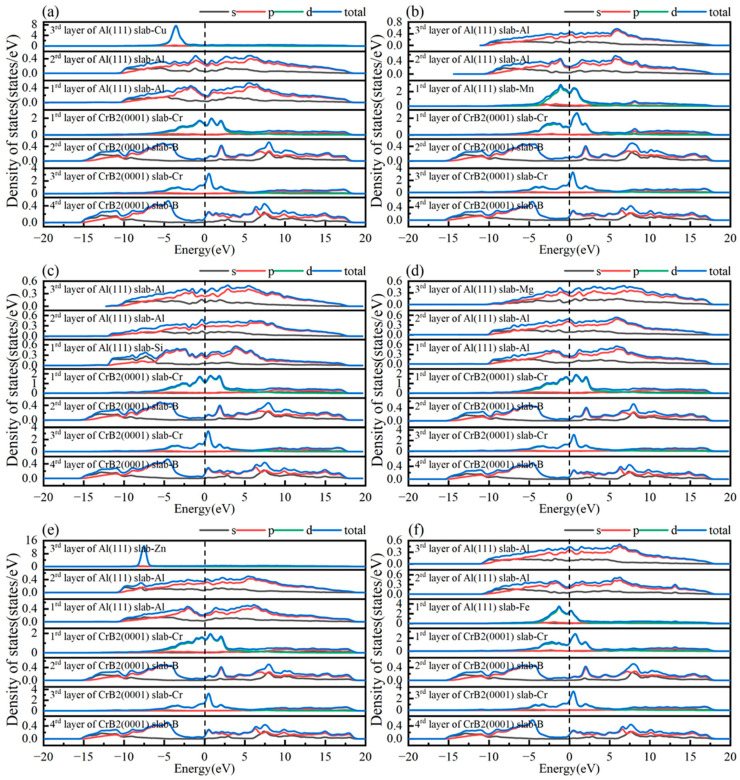
Density of state distributions for (**a**) Cu-doped interface, (**b**) Mn-doped interface, (**c**) Si-doped interface, (**d**) Mg-doped interface, (**e**) Zn-doped interface, and (**f**) Fe-doped interface.

To delve deeper into the bonding attributes of doped alloy atoms at the interface, a PDOS analysis was performed, as illustrated in Figure 10. Relative to the pristine interface shown in Figure 6a, interfaces doped with Mn and Fe display pronounced peak overlap and substantial hybridization effects. The interactions involving Mn-3d, Fe-3d, and Cr-3d orbitals at the interface are intensified. Together with the charge density difference analysis, this firmly establishes the formation of stable covalent bonds at the interface. In contrast, the PDOS curves for Cu-3d and Zn-3d orbitals appear sharp and focused, exhibiting characteristics markedly distinct from those of other layer atoms. At interfaces doped with Cu and Zn, the absence of significant peak overlap suggests a reduction in binding capacity post-doping, potentially compromising interface stability. This observation aligns with the insights gained from the charge density difference analysis. The PDOS profiles for Si- and Mg-doped interfaces largely mirror those of the undoped interface.

Among common alloying elements for aluminum, such as Cu, Mn, Si, Mg, Zn, and Fe, Mn and Fe contribute to enhancing the interfacial bonding strength and stability, thereby affecting the adhesion and overall performance of the coating. Thereby, the doping of Fe, Mn, and Mg may also help to improve the interfacial bonding strength and stability, while the doping of Cu, Zn, and Si may have a detrimental impact on interfacial stability.

## 4. Conclusions

This investigation utilizes density functional theory (DFT) to examine the structural characteristics of Al(111)/CrB_2_(0001) interfaces and assess how alloying components (Fe, Mg, Cu, Zn, Si, and Mn) affect interfacial bonding and electronic behavior. The analysis reveals several crucial observations:

1. The Cr-terminated configuration of the Al(111)/CrB_2_(0001) interface displays enhanced stability relative to alternative terminations, characterized by increased adhesive energy and reduced segregation enthalpy. Among various atomic arrangements, the hollow-site geometry presents the most favorable interfacial characteristics.
2. The adhesive energy of the Cr-terminated HCP-stacked Al(111)/CrB_2_(0001) interface exceeds that of the *α*-Al/Al boundary. This observation validates the potential of CrB_2_ particles as efficient substrates for *α*-Al grain nucleation, considering both structural and energetic aspects. The interface demonstrates pronounced covalent interactions, specifically involving Al-2p and Cr-3d orbital hybridization.
3. Alloying elements substantially modify Al(111)/CrB_2_(0001) interface behavior: (a) Fe, Mn, and Mg increase adhesive energy, facilitating nucleation within the aluminum matrix. (b) Cu, Zn, and Si reduce adhesive energy, possibly limiting CrB_2_ nucleation in aluminum. (c) The relative effectiveness of dopants, based on their influence on interfacial binding, follows the sequence Fe > Mn > Mg > Si > Zn > Cu. It is worth noting that the current findings are based on specific dopant configurations within the unit cell framework, where the substitution of one Al atom represents a high local dopant concentration. Further studies with larger supercells are recommended to validate trends under diluted conditions.
4. These results offer significant contributions to the development and refinement of Al-CrB_2_ composite materials, providing strategic directions for modifying interface characteristics through controlled alloying to improve material functionality across diverse applications.

## Figures and Tables

**Table 1:** Parameters used for calculations on the bulks, surface, and interface.

Phase	K-Point Meshes	Cutoff Energy/eV	Atom Number in Cell
Bulk Al(111)	10 × 10 × 10	500	4Al
Bulk CrB_2_(0001)	12 × 12 × 12	500	2B 1Cr
CrB_2_(0001)-Cr slab	16 × 16 × 1	500	8B 5Cr
CrB_2_(0001)-B slab	16 × 16 × 1	500	10B 4Cr
Al(111)/CrB_2_(0001)-Cr interface	18 × 18 × 1	500	8B 7Al 5Cr
Al(111)/CrB_2_(0001)-B interface	18 × 18 × 1	500	10B 7Al 4Cr

**Table 2:** The lattice parameters, unit cell volume, and formation enthalpy for bulk CrB_2_(0001) and Al(111) were computed and documented in this work.

Structures	Method	a = b	c	Volume (Å^3^)	ΔH_f_ (eV)
CrB_2_	GGA(PBE) [39]	2.977	2.962	22.73	−0.936
	Exp [40]	2.969	3.066	23.41	
	This work	2.959	3.032	22.99	−0.938
Al	GGA(PBE) [41]	4.05	4.05	66.43	
	Exp [42]	4.032	4.032	65.55	
	This work	4.049	4.049	66.38	0

**Table 3:** Interlayer distance perpendicular to the CrB_2_(0001) surface, in terms of absolute distance and as a percentage of the respective bulk spacing (%).

Termination	Interlayer	Slab Thickness (N)			
		5	7	9	11
Cr	Δ_12_	−14.38%	−18.35%	−14.52%	−15.86%
	Δ_23_	6.39%	5.53%	5.91%	6.67%
	Δ_34_		−4.51%	−4.06%	−3.67%
	Δ_45_			0.16%	1.83%
	Δ_56_				−0.15%
B	Δ_12_	6.78%	3.33%	5.68%	5.02%
	Δ_23_	−5.01%	−4.38%	−4.38%	−0.43%
	Δ_34_		−0.65%	0.50%	2.59%
	Δ_45_			−0.13%	0.98%
	Δ_56_				0.12%

**Table 4:** The surface energies of two different terminations of the CrB_2_(0001) slab with different numbers of layers.

Surface Energy (J/m^2^)					
Slab Thickness (N)		5	7	9	11
Termination	Cr	1.93	2.17	2.24	2.23
	B	1.89	2.15	2.18	2.17

**Table 5:** The interfacial separation and adhesive energy of the Al(111)/CrB_2_(0001) system were evaluated both prior to and following structural relaxation.

Termination	Stacking	Unrelaxed		Fully Relaxed		
		d_0_ (Å)	*W_ad_* (J/m^2^)	d_0_ (Å)	*W_ad_* (J/m^2^)	$\gamma_{\mathrm{int}}$ (J/m^2^)
Cr	OT	2.5	0.17	2.41	0.56	2.55
	MT	2.1	0.96	2.08	1.25	1.86
	HCP	2	1.15	2.05	1.75	1.31
B	OT	2.2	0.86	2.13	1.37	1.74
	MT	2	0.87	1.96	1.44	1.67
	HCP	1.7	0.96	1.58	1.56	1.55

**Table 6:** The total energy (eV), $W_{ad}$ (J/m^2^), and $\gamma_{\mathrm{int}}$ (J/m^2^) for different positions with doping elements.

Doping Element	Position	Energy (eV)	*W_ad_* (J/m^2^)	$\gamma_{\mathrm{int}}$ (J/m^2^)
Clean		−13,507.04	1.75	1.31
Cu	1	−15,076.33	1.26	2.48
	2	−15,076.86	1.55	1.90
	3	−15,076.98	1.67	1.77
Mn	1	−14,020.16	1.98	1.25
	2	−14,020.20	1.20	2.45
	3	−14,020.37	1.39	2.27
Si	1	−13,566.20	1.74	1.87
	2	−13,566.18	1.73	1.89
	3	−13,566.09	1.68	1.99
Mg	1	−15,085.85	1.16	1.71
	2	−15,086.32	1.84	1.23
	3	−15,086.38	1.90	1.17
Zn	1	−15,433.03	1.28	1.65
	2	−15,433.33	1.70	1.36
	3	−15,433.34	1.73	1.34
Fe	1	−14,257.71	2.15	1.16
	2	−14,257.94	1.44	2.15
	3	−14,258.07	1.54	1.99

## Data Availability

No new data were created or analyzed in this study. Data sharing is not applicable to this article.

## References

[1] Li X.R., Gao M.Q., Huan M., Li Y., Guan R.G. (2025). Microstructure and strength-conductivity synergy of Al-Mg-Si-Cu alloy sheets prepared via vacuum melting and thermo-mechanical treatment. J. Alloys Compd..

[2] Li W.H., Bai P.C., Cui X.M., Zhao X.P., Liang S.B., An J.L., Tian Y.J. (2024). In-situ TEM study on the precipitation behavior of an Al-Mn-Mg-Sc-Zr alloy formed by SLM. Mater. Charact..

[3] Li Y., Ma Z.P., Hao H.N., Zhu Y.K. (2024). First-principles analysis of the stability and electronic structure at the Al/Al4SiC4 interface. J. Mater. Heat Treat..

[4] Xiong C. (2018). Surface Electrospark Deposition of ZrB2-CrB2 Composite Coating for Spot Welding Electrodes. Master’s Thesis.

[5] Zhang S.S., Li X.W., Wang L., Zhang D., Yang P.L., Li Z.Y., Wang A.Y. (2016). The Influence of Deposition Temperature on the Structure and Properties of CrB_2_ Coatings. Chin. J. Surf. Eng..

[6] Zhang S.C., Wang Z.Y., Guo P., Ke P.L., Odén M., Wang A.Y. (2017). Temperature induced superhard CrB_2_ coatings with preferred (001) orientation deposited by DC magnetron sputtering technique. Surf. Coat. Technol..

[7] Wang Y.W., Sun X.W., Wang L., Yang Y., Ren X.X., Ma Y.D., Cui Y.H., Sun W.W., Wang X.Y., Dong Y.C. (2021). Microstructure and properties of CrB_2_-Cr_3_C_2_ composite coatings prepared by plasma spraying. Surf. Coat. Technol..

[8] Choi H.S., Park B., Lee J.J. (2007). CrB_2_ coatings deposited by inductively coupled plasma assisted DC magnetron sputtering. Surf. Coat. Technol..

[9] Audronis M., Rosli Z.M., Leyland A., Kelly P.J., Matthews A. (2008). Tribological behaviour of pulsed magnetron sputtered CrB_2_ coatings examined by reciprocating sliding wear testing against aluminium alloy and steel. Surf. Coat. Technol..

[10] Morgiel J., Poliarus O., Pomorska M., Maj Ł., Szlezynger M. (2020). Thermal stability of plasma-sprayed NiAl/CrB_2_ composite coatings investigated through in-situ TEM heating experiment. Mater. Charact..

[11] Zhuo Z.M. (2020). Research on the Micro-Scale Interface Structure of Niobium-Based Heterocore with Aluminum Melt. Ph.D. Thesis.

[12] Saengdeejing A., Sahara R., Toda Y. (2024). Al–Ni–Ti thermodynamic database from first-principles calculations. Calphad.

[13] Pang X.Z., Yang J.B., Li A., Pang M.J., Xiao Y., Nong H., Qin H.Q., Liu C.Y. (2023). Understanding the atomic and electronic structure of the NbC(111)/Cu(111) interface via first principles calculation. Mater. Today Commun..

[14] Song Y.L., Wang G.C., Ni J.J., Song B., Guo S., Li X.X., Cheng C. (2024). First-principles study on the structural, mechanical and thermodynamic properties of Cu-Cr-Zr alloy. Phys. B Condens. Matter.

[15] Yang Z.C., Cheng L.X., Chen S.Z., Zhang Y. (2024). Active element Ti improves the Sn-based alloy filler/graphite soldering interface: A combined first-principles and experimental study. Mater. Sci. Semicond. Process..

[16] Zou X., Liu T.Y., Liu W.H., Li Y.M., Zhao Y.P. (2024). First principles calculations on stability, electronic structure and fracture failure of Cu-doped Al(100)/Mg_2_Si(111) interface. Mater. Chem. Phys..

[17] Han F., Li C., Wang Y., Pai Z., Meng Y., Cao M., Liu Y., He P., Ma X., Xue L. (2024). Comparative study on corrosion property of 2219 aluminum alloy sheet and additively manufactured 2319 aluminum alloy. J. Mater. Res. Technol..

[18] Wu X., Guan Z.P., Yang H.Y., Dong B.X., Zhang L.C., Meng J., Luo C.J., Wang C.G., Cao K., Qiao J. (2024). Sub-rapid solidification microstructure characteristics and control mechanisms of twin-roll cast aluminum alloys: A review. J. Mater. Res. Technol..

[19] Wang Z.X., Zhan L.L., Yun X. (2024). Experimental study of local buckling behaviour of 7A04-T6 high strength aluminium alloy H-section stub columns in fire. Eng. Struct..

[20] You X., Xing Z., Jiang S., Zhu Y., Lin Y., Qiu H., Nie R., Yang J., Hui D., Chen W. (2024). A review of research on aluminum alloy materials in structural engineering. Dev. Built Environ..

[21] Yin J.B., Lu X.F. (2018). Theoretical study of electronic structure and optical properties of tin doped CuS counter electrode for dye-sensitized solar cells. Sol. Energy.

[22] Nazmul H., Mehedi H.M., Alamgir K., Harunur R.M. (2023). Theoretical study of the structural, electronic, mechanical, and optical of transition metal (mn, co, and ni) doped FrGeI_3_ perovskites. Results Mater..

[23] Dong D., Kuang X.Y., Guo J.J., Zheng B.X. (2010). Density functional theory study of AunMn (n = 1–8) clusters. J. Phys. Chem. Solids.

[24] Ganeshan S., Hector L.G., Liu Z.-K. (2011). First-principles calculations of impurity diffusion coefficients in dilute Mg alloys using the 8-frequency model. Acta Mater..

[25] Ivashchenko V.I., Onoprienko A.A., Skrynskyy P.L., Kozak A.O., Vedel D.V., Mazur P.V., Sinelnichenko A.K., Buranych V.V., Pogrebnjak A.D. (2024). Structure and properties of (TiZrHfNbTa)B2 films and first-principles models for high entropy diborides. Thin Solid Film..

[26] Zhu H., Wang Q., Yang C., Wang Y., Xia C., Zhao D., Zhang H., Wang M., Chen Z., Wang H. (2024). Improving TiB_2_ dispersion in Al-Si composites by interfacial projection: High-throughput first-principles calculations and experimental verification. Mater. Des..

[27] Zhang X.Q., Yue Y.L., Xu D., Qin J.Q., Zhang X.Y., Liu R.P. (2024). Effect of solutes segregation in a binary TiAl alloy: A first-principles calculation method. Mater. Today Commun..

[28] Segall M.D., Lindan P.J.D., Probert M.J., Pickard C.J., Hasnip P.J., Clark S.J., Payne M.C. (2002). First-principles simulation: Ideas, illustrations and the CASTEP code. J. Phys. Condens. Matter.

[29] Perdew J.P., Burke K., Ernzerhof M. (1996). Generalized gradient approximation made simple. Phys. Rev. Lett..

[30] Fischer T.H., Almlöf J. (1996). General methods for geometry and wave function optimization. J. Phys. Chem..

[31] Ji L.G., Liu H.L., Huang C.Z., Tang Y.Q., Huang J., Qiu Y.H. (2024). Effect of Al and Si content on properties of Ti_(1-x-y)_Al_x_Si_y_N coating materials: First-principles calculation. Mater. Today Commun..

[32] Liu X., Jiang X., Wang T., Zhang Z., Liu Z. (2023). Lattice thermal conductivity of two-dimensional CrB_4_ and MoB_4_ monolayers against Slack’s guideline. Results Phys..

[33] Wang J.H., Hong W., Liu T.Y., Lu X., Li H.F. (2024). The electronic and optical properties of Al interstitial defects in KH2PO4 crystal: First principles study. Comput. Mater. Sci..

[34] Gao D.L., Yi D., Xia J., Yang Y.G., Wang X. (2024). First-principles screening of Cu-based single-atom alloys for highly efficient electrocatalytic nitrogen reduction. Mol. Catal..

[35] Sun M.Y., Mao H., Xu D.J., Zhou G.X., Li J.B., Gong H.R., Liang C.P. (2023). First-principles calculation of various phase transition in Al–Sc system. J. Mater. Res. Technol..

[36] Weng J.H. (2019). Preparation and Property Study of Superhard and Corrosion-Resistant CrB2 Coating. Master’s Thesis.

[37] Bramfitt B.L. (1970). The effect of carbide and nitride additions on the heterogeneous nucleation behavior of liquid iron. Metall. Mater. Trans. B.

[38] Qin N. (2011). The First Principles Study of the Stability for Transition Metal Diborides (0001)Surfaces. Master’s Thesis.

[39] Sun Y. (2016). First-Principles Calculation of Phase Stability and Physical Properties of Transition Metal Borides. Master’s Thesis.

[40] Zhang S.S. (2016). Preparation and Study of Structure and Properties of Chromium Diboride Coating.

[41] Besson R., Macaluso S., Thuinet L. (2022). Critical issues on coherent interface energy calculations revisited: The case of Al/TiB_2_. Surf. Interfaces.

[42] Zhang S., He D., Huang P., Wang F. (2021). Moiré pattern at graphene/Al(111) interface: Experiment and simulation. Mater. Des..

[43] Xin T., Bai L.J., Chen G.T., Zhang G.J., Zhao B. (2024). Fe/Cu doped black ceramic coating on magnesium alloy by plasma electrolytic oxidation method based on first principles. Surf. Interfaces.

[44] Zunger A., Malyi O.I. (2021). Understanding doping of quantum materials. Chem. Rev..

[45] Wang M.J., Sun J.W., Li S.Y., Meng Y.C., Zheng H.Y., Yin Z., Fu Y.Z., Zhang Y.J. (2024). Heterogeneous nucleation mechanisms in Mg(0001)/ Al_3_BC(0001) interfaces: Insights for advanced Mg-based composites. Surf. Interfaces.

[46] Pei X., Yuan M.N., Han F.Z., Wei Z.Y., Ma J., Wang H.L., Shen X.Q., Zhou X.S. (2022). Investigation on tensile properties and failure mechanism of Al(111)/Al_3_Ti(112) interface using the first-principles method. Vacuum.

[47] Wang M.J., Han H.M., Zhang G.W., Xu H., Yin Z., Dong Y., Fu Y.Z. (2022). Effect of solute elements (Ni, Sn, P) on the adhesion electronic properties of γ-Fe/Cu heterogeneous interface: A first-principles study. Results Phys..

[48] Wang M., Wei D., Lai Y., Shou H., Liu S., Zheng H., Zhang Y., Yang X., Zhao T., Wang R. (2024). Enhanced nucleation at Al(111)/Ti_3_AlC_2_(0001) interfaces: The role of doping in adhesion and interfacial stability. Vacuum.

[49] Dai Y.C., Liu J., Shi Y. (2025). Effects of transition elements additions on interfacial properties of Al(111)/B_4_C (0001) interface based on first-principles study. J. Phys. Chem. Solids.

